# Cascading effects of moth outbreaks on subarctic soil food webs

**DOI:** 10.1038/s41598-021-94227-z

**Published:** 2021-07-23

**Authors:** Irene Calderón-Sanou, Tamara Münkemüller, Lucie Zinger, Heidy Schimann, Nigel Gilles Yoccoz, Ludovic Gielly, Arnaud Foulquier, Mickael Hedde, Marc Ohlmann, Mélanie Roy, Sara Si-Moussi, Wilfried Thuiller

**Affiliations:** 1 Univ. Grenoble Alpes, Univ. Savoie Mont Blanc, CNRS, LECA, Laboratoire d’Ecologie Alpine, 38000 Grenoble, France; 2 Institut de Biologie de L’ENS (IBENS), Département de biologie, École Normale Supérieure, CNRS, INSERM, Université PSL, 75005 Paris, France; 3 INRA EcoFoG (AgroParisTech, CNRS, CIRAD, INRA, Université Des Antilles, Université de Guyane), Kourou, France; 4 Department of Arctic and Marine Biology, UiT The Arctic University of Norway, Tromsø, Norway; 5 Eco&Sols, Univ Montpellier, CIRAD, INRA, IRD, Montpellier SupAgro, 34398 Montpellier, France; 6 Université Savoie Mont-Blanc, LAMA, 73000 Chambéry, France; 7 Laboratoire Évolution Et Diversité Biologique, CNRS, UMR 5174 UPS CNRS IRD, Université Toulouse 3 Paul Sabatier, Toulouse, France; 8 Instituto Franco-Argentino Para El Estudio del Clima Y Sus Impactos (UMI IFAECI/CNRS-CONICET-UBA-IRD), Dpto. de Ciencias de La Atmosfera Y Los Oceanos, FCEN, Universidad de Buenos Aires, Intendente Guiraldes 2160 - Ciudad Universitaria (C1428EGA), Ciudad Autónoma de Buenos Aires, Argentina

**Keywords:** Biodiversity, Ecological networks, Ecosystem ecology

## Abstract

The increasing severity and frequency of natural disturbances requires a better understanding of their effects on all compartments of biodiversity. In Northern Fennoscandia, recent large-scale moth outbreaks have led to an abrupt change in plant communities from birch forests dominated by dwarf shrubs to grass-dominated systems. However, the indirect effects on the belowground compartment remained unclear. Here, we combined eDNA surveys of multiple trophic groups with network analyses to demonstrate that moth defoliation has far-reaching consequences on soil food webs. Following this disturbance, diversity and relative abundance of certain trophic groups declined (e.g., ectomycorrhizal fungi), while many others expanded (e.g., bacterivores and omnivores) making soil food webs more diverse and structurally different. Overall, the direct and indirect consequences of moth outbreaks increased belowground diversity at different trophic levels. Our results highlight that a holistic view of ecosystems improves our understanding of cascading effects of major disturbances on soil food webs.

## Introduction

Natural disturbances, such as fires, droughts, or insect outbreaks, are key drivers of ecosystem dynamics and community structure^1^. Global change could exacerbate their severity and frequency worldwide with potential extensive impacts on biodiversity, ecosystems and human societies^2,3^. Understanding the effect of disturbances on the dynamics and structure of biodiversity is therefore more than ever a crucial issue in ecology. Yet, the high variability of local biodiversity trends in response to global changes asks for more integrative analyses, going beyond mere measures of species richness and accounting for the multiple components of the ecosystems^4,5^. Particularly, soil organisms are rarely included when synthesizing biodiversity trends in the face of disturbances, despite their recognized and well documented influence on multiple ecosystem functions (e.g. nutrient cycling) and nature contributions to people (e.g. carbon storage or depollution)^6–9^.

Most studies quantifying the effect of disturbances on biodiversity have focused on a single trophic or taxonomic group, often directly affected by the disturbance, like plants^9^. However, much less is known on how the effects propagate across trophic levels ultimately affecting the entire ecosystem. Plants and soil organisms are tightly linked through direct and indirect interactions, including mutualism, parasitism or predation, which promote the exchange and supply of nutrients and ensure multiple ecosystem processes^6,7^. Ignoring these trophic interactions and how resource deprivation in one trophic level can cascade to other levels may obscure the true consequences of disturbances for ecosystems^10^. Furthermore, misleading conclusions could be drawn if resulting disturbance effects differ between trophic levels^11^. Most natural disturbances cause immediate fluctuations in the quantity and quality of available soil resources^1^. Extreme winds can remove or deposit organic matter on the forest floor, while insect outbreaks increase soil nutrient inputs through defoliation and insect faeces and corpses. These local changes in basal resource availability can have important consequences on the abundance and diversity of primary producers (e.g. plants or nitrifying bacteria) and primary consumers (e.g. decomposers or herbivores), but also subsequently on the whole soil food web through bottom-up cascading effects^12–14^. Predicting whether the effects would vanish or amplify remains challenging due to the complexity of soil food webs in real ecosystems. Stoichiometry-based studies have provided numerous evidences that such indirect effects propagate across soil food webs from the microfauna to the macrofauna in terms of composition and biomass^15–17^. However, these approaches don’t include the microbial part of the soil food web, and often lack resolution or breadth for the micro and macro fauna when describing the diversity and composition of these complex communities. In addition, changes in the abundance and diversity of organisms across the food web are likely to induce structural changes in the entire interaction network, potentially leading to alternative ecosystem states^8,18,19^. Thus, quantifying cascading effects of disturbances on ecosystems requires a holistic view of biodiversity with not only exhaustive sampling methods to capture all-in-end biodiversity, but also a suitable analytic approach to analyze changes in trophic levels and interactions.

To meet this challenge, we combined the power of environmental DNA metabarcoding (eDNA)^20^ to obtain a nearly complete view of the belowground biodiversity, with a food web approach and network theory. Grouping species with the same trophic position (i.e. shared predators and preys/resources) in ecological networks facilitates the study of complex multitrophic communities^21–23^. In such an approach, the focus is not on species, but rather on trophic groups and trophic interactions. The definition of the trophic groups depends both on the resolution of the observation units (e.g. the taxonomic resolution) and the information available on their diet or trophic position^24–26^, and is also related to the ecological question. When studying the large-scale consequences of disturbances on biodiversity, there is a trade-off between sufficiently fine resolution to reliably and meaningfully measure cascading effects^22,27^, and sufficiently broad resolution to avoid knowledge gaps and cope with heterogeneity of taxonomic resolution in the data^25,28^. Once a food web is built, diversity can be measured within trophic groups (e.g. species diversity) and between trophic groups (e.g. trophic diversity or diversity of interactions), allowing the integration of ecological processes occurring at different dimensions of the food web (e.g. competition and predation)^29,30^. For this, network theory provides appropriate metrics to describe and compare the diversity and structure of ecological networks, accounting for both group abundances and interactions^31,32^.

**Figure 1:**
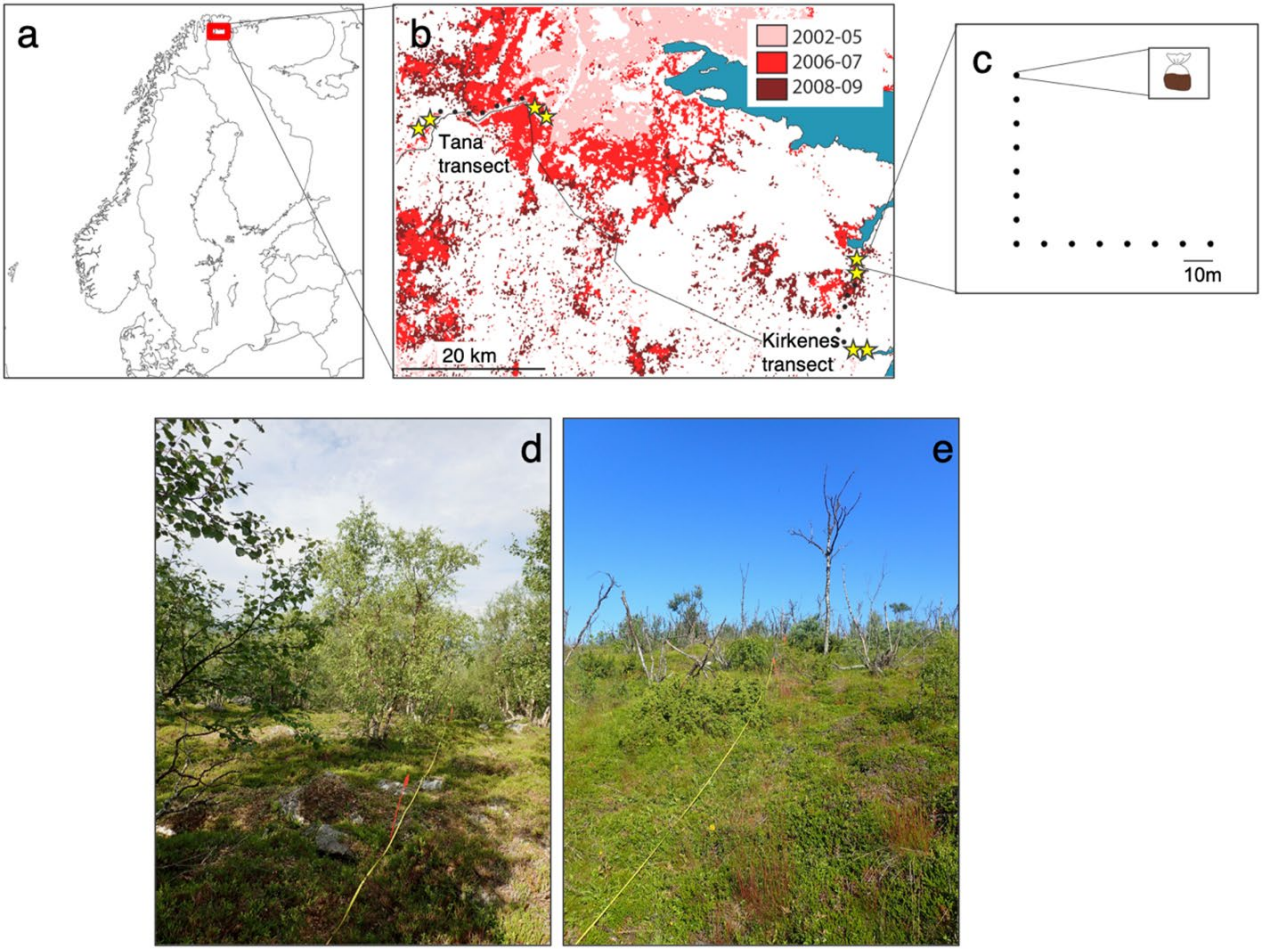
Sampling design in undamaged and defoliated forests. Map of the study location in the Northeastern Norway (**a**), in the Varanger region (**b**). Red areas (**b**) represent birch forest that experienced severe defoliation during the most recent moth outbreak. Yellow stars indicate the stations that were sampled in this study, at each extreme of two pre-established transects (black dots) going from highly defoliated forests stands to undamaged stands. Soil sampling was conducted in each station along an L-shaped transect (**c**). Photos illustrate the stations from undamaged (**d**) and defoliated (**e**) forests. Red flags in the photos indicate the sampling points represented in (**c**). Undamaged forests were characterized by living birch trees (*Betula pubescens*) and a shaded understory dominated by ericaceous shrubs (e.g. *Empetrum nigrum*). Defoliated forests were characterized by dead birch trees, patches of remaining ericaceous shrubs and a soil covered by light-tolerant grass and herbs including the dominant *Avenella flexuosa*. Photo credits: Heidy Schimann. Map (**a**) was created using ArcGIS® software 10.4.1 by Esri (www.esri.com). Map (**b**) was modified from^38^ (https://doi.org/10.1371/journal.pone.0099624.g001).

Here, we study the effect of moth outbreaks on soil food webs of subarctic birch forests in Northern Fennoscandia. These forests have experienced moth outbreaks of unprecedented scale and severity in recent decades, which have led to a sudden and persistent vegetation change -from birch forests with understory dominated by dwarf shrubs to grass-dominated systems associated with high tree mortality- that was still visible 8 years after the disturbance^33–36^ (Fig. 1). Moth outbreaks is a good model for assessing the cascading effects of disturbance on soil food webs, as the larvae only attack the foliage of the dominant primary producers, i.e. the birch tree (*Betula pubescens*), and some abundant species of erect and dwarf shrubs in the understory layer (e.g. *Betula nana, Empetrum nigrum, Vaccinium* spp*.*). In parallel, soil organic matter is enriched through dead plants and N addition from larval faeces and corpses^37,38^. We can therefore expect that impacts on the whole soil food web arise from bottom-up effects from changes in the vegetation and basal resources to the other trophic compartments^12^. Drastic shifts in the composition of biological communities following defoliation have been already reported in these nutrient-limited soils where the dominance of the allelopathic dwarf shrub *Empetrum nigrum* in the understory leads to regressive succession that may inhibit soil microbial activity, organic matter decomposition, and thus nutrient availability^39–41^. These shifts correspond to a replacement of *Empetrum nigrum* by the grass *Avenella flexuosa*^34^ with subsequent effects on the diversity and abundance of organisms directly relying on plants, including vertebrate herbivores^33^, birds^42^, saproxylic beetles^38^, and fungal communities^43,44^. However, we still ignore whether moth outbreaks induced indirect effects across the soil food web, whether these effects are of comparable magnitude to those observed for vegetation, and finally, whether these effects have significant consequences on trophic interactions and ultimately on the whole soil food web structure.

**Figure 2:**
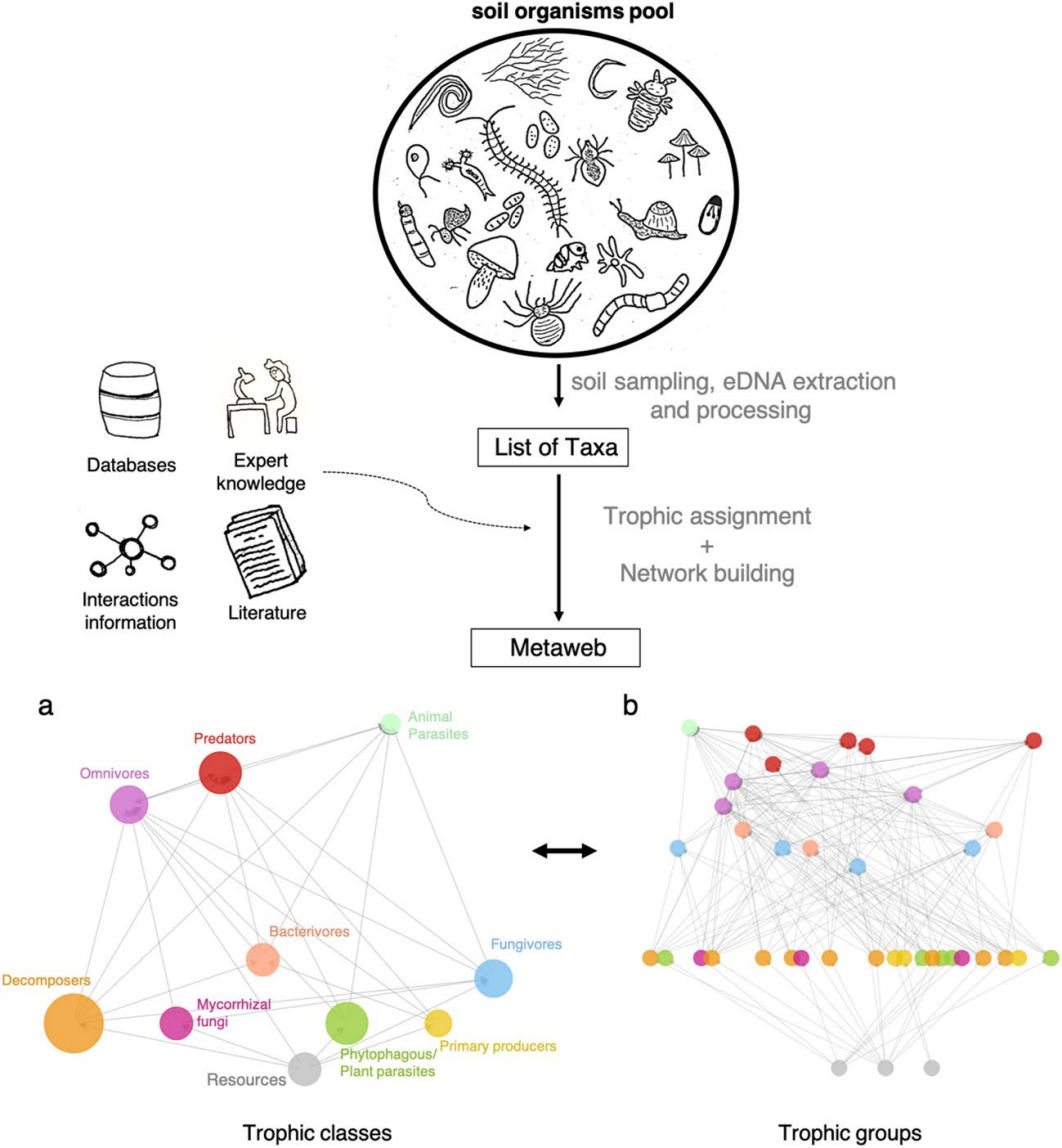
Methodology used to build the *metaweb* from soil eDNA. First, eDNA was extracted and processed from the 86 soil samples to obtain a list of taxa for the study area. Second, using an extensive collection of trophic knowledge from databases, literature and experts, taxa were assigned to broad trophic classes and then to finer trophic groups, which separate distant phylogenetic groups or groups that differ in their resources acquisition strategy. Main trophic links were collected from literature and current knowledge to build the *metaweb* at two levels of resolution (**a**,**b**). The colours correspond to the trophic classes (**a**) that are refined and split in the highly resolved *metaweb* (**b**).

We used eDNA data obtained from 86 soil samples from two well-studied areas in northeastern Norway (i.e., Tana and Kirkenes). This study design allowed for appropriate pairwise comparisons between coupled undamaged and defoliated forest based on well-documented defoliation patterns from both remote sensing and field methods (Fig. 1). The sampling design aimed at capturing the environmental heterogeneity at different spatial scales of the landscape within these areas. We then classified both microorganisms and macroinvertebrates into 9 broad trophic classes and 37 finer trophic groups to build metawebs^45^ at two levels of resolution for the study area (Fig. 2). The metawebs were then used to infer local soil food webs based on taxa detected locally in each soil sample. The trophic class resolution corresponds to what is commonly used in soil food web ecology (e.g.^22,27^), but we additionally included the trophic group resolution because a finer resolution is needed to capture specific effects of disturbance on groups that are hidden at a coarser resolution. For instance, different types of mycorrhizal fungi like arbuscular mycorrhizal fungi and ectomycorrhizal fungi may have opposite responses to tree defoliation, the former increasing and the later decreasing in their proportion following disturbances^46^.

Using this approach, we tested three hypotheses about the cascading effects of moth defoliation on the local soil food webs at different levels of organization. First, (*H*_*1*_) moth defoliation changes the diversity in MOTUs (Molecular Operational Taxonomic Unit) and the relative abundances of most trophic groups. We expected positive effects on most decomposers and their consumers through the impulse in soil resources availability^47,48^ from both moth outbreaks and the decreased abundance of the allelopathic species *Empetrum nigrum*. In parallel, we expected negative effects on e.g., ectomycorrhizal and ericoid mycorrhizal fungi, as the result of the decline of birch and ericaceous shrub roots. Second, (*H*_*2*_) the magnitude of the effect differs among trophic groups across the soil food web. We expected the effect of defoliation to be stronger for primary consumers and decomposers that are directly affected by changes in basal resources availability and plant composition, and then to decrease toward higher trophic levels (attenuation of the effects). Third, (*H*_*3*_), moth defoliation changes the overall structure of the local soil food webs^10,49^. We expected to observe differences in the trophic groups and links diversity and composition of the local food webs between defoliated and undamaged forests.

## Results and discussion

**Figure 3:**
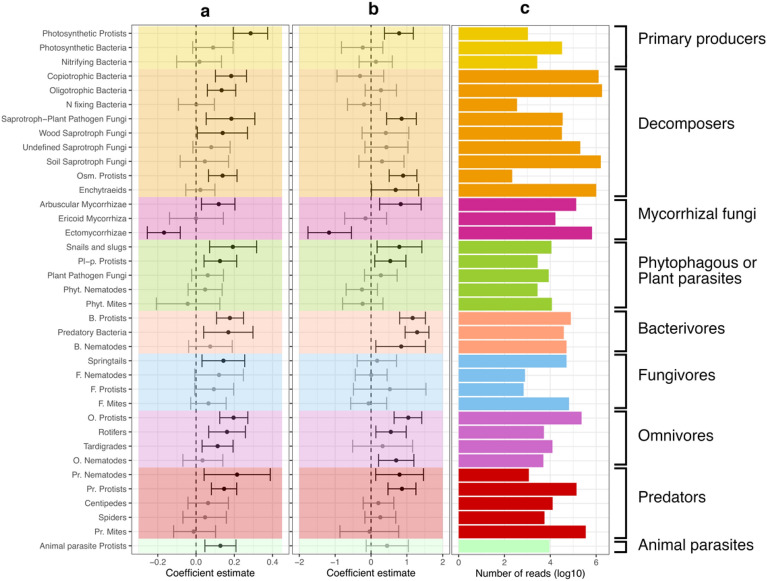
Effect of defoliation on diversity and relative abundance of trophic groups. Effect size of defoliation on MOTU diversity (**a**) and relative abundance of reads (**b**) for each trophic group with 90% credible intervals. The barplot (**c**) shows the total number of reads (logarithmic scale) of each trophic group in the overall dataset. A multilevel linear model was fitted individually for each trophic group with a dummy variable for defoliation as predictor and a random factor accounting for the nested sampling design. MOTU diversity was standardized by the maximum value observed within each trophic group to obtain comparable effect sizes between groups. The colours correspond to the trophic class definitions (see Fig. 2).

Fitting a multilevel linear model for each trophic group, we found that moth defoliation increased MOTU diversity and the relative abundances of most trophic groups (Fig. 3). This is consistent with *H*_*1*_ and food web theory predictions, i.e. the effect of disturbances should propagate up the food web levels when resources are enriched through bottom-up processes^14,50^. Overall, diversity and relative abundance followed similar trends within trophic groups (Fig. 3a,b).

The basal groups directly linked to plants or basal resources (e.g., soil organic matter and light), i.e., mycorrhizal fungi, phytophagous or plant parasites, decomposers and primary producers, were expected to respond to changes in the composition of plant communities and nutrient enrichment following the outbreak. Here, comparing undamaged and defoliated forests, we observed a radical shift from ectomycorrhizal to arbuscular mycorrhizal fungal communities. This is consistent with the reduction of birch fine woody roots in defoliated forests, which are obligate hosts for most ectomycorrhizal fungi, and with the increases of herb and grass roots that are mostly associated with arbuscular mycorrhizal fungi^43,44,51^ (Supplementary Fig. 2). The increased diversity and relative abundance of slugs, snails and plant pathogen protists could be in part explained by the increased palatability of the plant assemblages. Indeed, grasses like *Avenella flexuosa,* which is dominant in the defoliated forests, are more palatable as compared to allelopathic species like *Empetrum nigrum*^33,52^. Photosynthetic protists diversity and relative abundance also increased in defoliated sites which are more open, hence allowing more light to reach the soil (Fig. 1).

Among the decomposers, defoliation led to an increase in the diversity of heterotrophic bacteria, protists, saprotroph-plant pathogen fungi and wood saprotroph fungi. Similarly, the relative abundance of protists, saprotroph-plant pathogen fungi and enchytraeids increased. Differences in plant litter chemistry between undamaged and defoliated forests (Supplementary Fig. 2) might drive the communities of decomposers^53^ and could explain these changes. For instance, the litter produced by *Empetrum nigrum,* which dominates undamaged forests, releases of phenolic compounds^52^ that can strongly reduce plant species diversity^40,41^. Such detrimental effects might also hold true for the diversity and abundance of most decomposers. Soils from defoliated forests had lower C/N ratios, suggesting that defoliation promote more labile, easily decomposable organic matter inputs (Supplementary Fig. 3) but more precise soil nutrient measurements would be needed to confirm this.

Contrary to our expectation, the magnitude of the effect of defoliation did not decrease further up the food web (Fig. 3a,b), but was instead equally important at all trophic levels. This result did not depend on the number of sequences obtained for each group (Fig. 3c). This rejects the hypothesis of a mitigation of the effects of the disturbance when moving up to higher trophic levels in the soil food web (*H*_*2*_). For example, the indirect effect of defoliation on the diversity of copiotrophic bacteria was as strong as the effect on their protist predators, and as strong as the effect on nematodes feeding on protists. In addition, the effect of defoliation on animal parasites, which are at the top of the soil food web, was similar to the effect on mycorrhizal fungi. Our findings are consistent with other studies pointing out that species-poor ecosystems, like subarctic birch forests, could be more prone to the propagation of bottom-up disturbances along food webs^54^. Furthermore, while some groups were affected by defoliation, other groups within the same trophic class were not (e.g. herbivore mite *vs.* plant pathogens protists, or ectomycorrhizal *vs.* ericoid mycorrhizal fungi). Other studies have highlighted the challenge of predicting the effect of an environmental stressor on overall biodiversity due to the variety of responses that organisms can have, associated with attributes such as dispersal abilities or resistance structures (e.g. cysts in protists)^55,56^. This is particularly important in soil food webs consisting of organisms with large differences in body size, life-span and life history strategies, and therefore in their response time to disturbance, which can vary from seconds to decades^7,48,56^. This complexity hampers our ability to detect consistent patterns when studying soil food webs at fixed sampling times.

**Figure 4:**
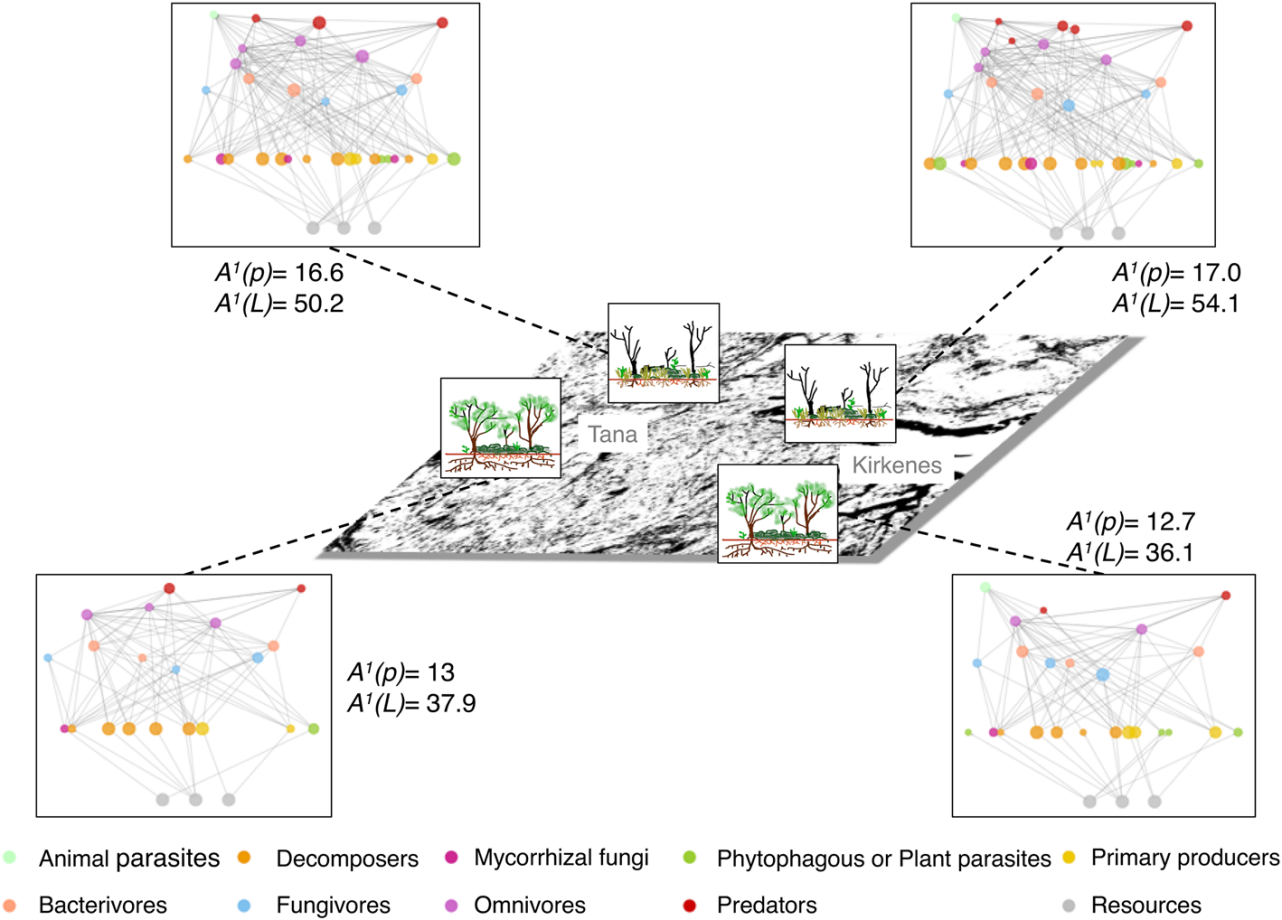
Topology and α-diversity of local food webs in undamaged *vs.* defoliated forests. The values represent the α-diversity of the soil food webs for each area and category of defoliation at the trophic group resolution: A^1^(**p**) is the diversity in trophic group abundances (nodes) and A^1^(**L**) the diversity in trophic links abundances (edges) using Shannon diversity. Nodes of the local food webs corresponded to the local relative abundances of the groups varying from 0 (when the group was absent) to 1 (when the group was at its maximum observed abundance). Links were binary links (i.e. present or absent) assuming an interaction when the two groups concerned were present. For the visualization, four local soil food webs (with an average value of A^1^(**p**)) were selected to highlight the differences in diversity between undamaged and defoliated forests of each area. The colours correspond to the trophic classes and the nodes are distributed vertically based on their trophic level from the bottom (basal levels) to the top (higher levels).

**Figure 5:**
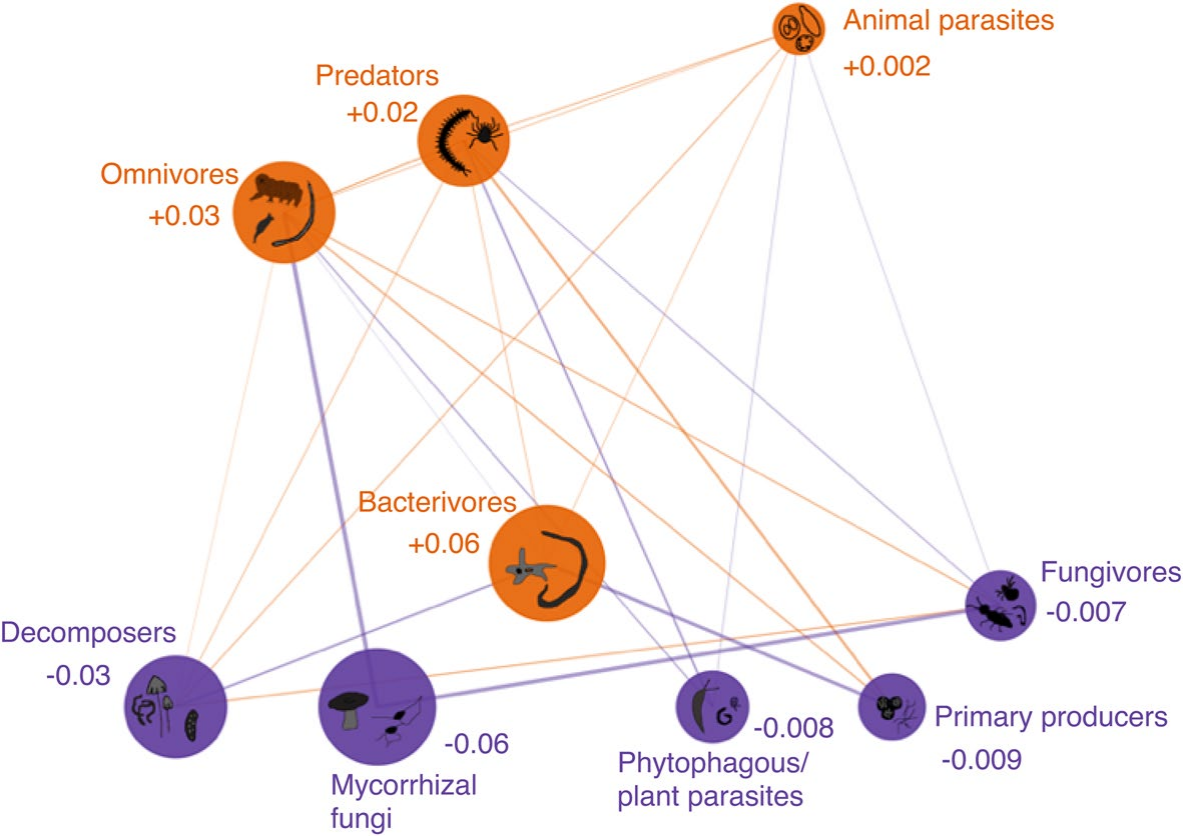
Structural differences among the local soil food webs from undamaged to defoliated forests at the trophic class resolution. Orange colour represents an increase, and purple colour a decrease in the relative abundance proportion within the local food webs of trophic classes (nodes) and link probability between classes (edges) from undamaged to defoliated forest. Relative abundance proportion corresponds to the sum of the relative abundances of the trophic groups inside the trophic class normalized within the local food web to sum one. Link probability corresponds to the probability of interaction between two classes given the links between their respective trophic groups and the relative abundances of these groups. The widths of the edges are scaled by the square root of the changes in link probability. Size of the nodes are proportional to the value of change in relative abundances proportion within the soil food web, indicated with numbers. Nodes are distributed vertically based on their trophic level from the bottom (basal levels) to the top (higher levels).

We then examined how changes in trophic groups relative abundances influenced the network structure of local soil food webs, using network diversity indices^31^. Following *H*_*3*_, moth defoliation significantly altered the whole soil food web structure in terms of node and link abundances, both for the trophic class and group resolutions (Supplementary Fig. 4). An increase in local diversity (α-diversity) of trophic groups and links in defoliated forests partially explained the changes in food web structure (Fig. 4). When zooming out to trophic classes, differences in the α-diversity of soil food webs were less obvious but food webs were nevertheless slightly more diverse for defoliated forests (Supplementary Fig. 5). This reflects that within a trophic class, trophic groups can have opposite responses (Fig. 3) that are averaged out when only considering trophic class, and highlights the importance of using a finer trophic resolution than what is often used in the literature to understand the variability of cascading effects in the different components of the soil food web. On average, we observed a decrease in the proportion of most classes of primary consumers (i.e. plant mycorrhiza, herbivores/plant pathogens, decomposers) within the soil food webs in defoliated forests, and an increase in the proportion of higher trophic level classes (i.e. bacterivores, omnivores, predators), which were rare in the undamaged forests (Fig. 5). These changes in relative abundance proportions within the soil food web are not to be confounded with the individual changes in the relative abundances of the trophic groups (Fig. 3b). For instance, a decrease in the proportion of some classes might be related to weaker increase in average of the relative abundance of the groups within the class from undamaged to defoliated forests, compared to a stronger average increase for classes in higher trophic levels.

The observed shifts in the structure of soil food webs could translate into impacts on multiple ecosystem functions, including carbon and nutrient fluxes, and plant productivity^21,22^. Recent studies have observed a slowdown in soil C and N cycles following severe outbreaks in these forests and have related this result to the decrease in the below-ground C-allocation to the rhizosphere and the decrease of ectomycorrhizal fungi^37,51^. An interesting avenue would be to relate how other components of the food web diversity (e.g. decomposer channel) contribute to the C:N stoichiometry to derive predictions on the long-term effects of these important disturbances on biogeochemical cycles.

The spatial extent of the study was limited to two landscape areas of ca 20 km extent, and we acknowledge that further monitoring would be required to assess the full extent of soil food webs responses to moth outbreaks in subarctic birch forests. Previous studies have found that the effect of moth outbreaks on biological communities can vary depending on local productivity and climatic conditions (as represented by the two areas Tana and Kirkenes)^33,34,42^. We found, however, a consistent response for most soil organisms across the two areas that translated into significant local changes in the whole soil food web diversity and composition. The consistency and strength of the effects of defoliation on the different facets of local soil food webs point to general conclusions on the bottom-up cascading effects of moth outbreaks on soil communities in these subarctic birch forests, despite the heterogeneity in environmental condition of the studied system.

## Conclusion

The multitrophic approach used in this study, which combines an exhaustive diversity sampling (here eDNA data) with current trophic knowledge, an extended soil food web approach and ecological network theory, allows understanding the cascading effects of disturbances on soil biodiversity. We demonstrated that recent moth outbreaks in birch forests of Northern Fennoscandia caused major local shifts in the diversity and relative abundance of most trophic groups, ultimately changing the structure of the soil food web. We found more diverse soil food webs in defoliated forests compared to undamaged forests, accompanied by an increase in the proportion of groups in higher trophic levels.

We emphasize the need to consider different levels of resolution to ensure the robustness of conclusions and improve our understanding of how soil diversity responds to disturbances. Highly resolved food webs allow to map the cascading effects by revealing the variability of organisms’ responses. In contrast, low resolution food webs provide a general picture on how these changes affect the food web structure. Our study opens new prospects in understanding the response of complex and diverse food webs to disturbance.

## Material and methods

### Sampling

The study took place in the Varanger region at approximately 70° N, 29° E, Northeastern Norway. This region is located in the transition between subarctic deciduous forests and the arctic tundra. Periodic outbreaks of the autumn moth (*Epirrita autumnata*) and more recently the winter moth (*Operophtera brumata*) have occurred in the region with a 9–10-years frequency approximately. Recently, the consecutive episodes by the two species caused a severe mortality of birch trees^35^. Sampling was replicated in two areas located approximately 70 km apart, both at the border of the outbreak range, but with slight differences in the defoliation year: Tana (70°03′ N, 27°45′ E.), defoliated during 2006–2007, and Kirkenes (69°46′ N, 29°20′ E) defoliated during 2007–2009. Differences in the forest characteristics between these two areas allow to control for the influence of the initial forest characteristic on the effect of defoliation, that has been proved to be non-negligible in past studies^34,38,42^. In each area, stations along a linear transect were previously established from highly impacted forest stands to undamaged stands^38,42^. In order to maximise the differences between defoliated and undamaged forests we selected the two stations at one extreme of the transect corresponding to defoliated forest, i.e. almost all tree stems dead or heavily damaged, and the two stations at the other extreme of the transect corresponding to undamaged forest, i.e. all trees alive, based on the damage-scores measured in^38,42^ (Fig. 1). The two adjacent stations, separated from at least 2 km within defoliated or undamaged forests, were considered as local replicates and were surrounded by a large area of forest in their same condition, i.e. defoliated or undamaged. Defoliated and undamaged stations within an area were ca. 20 km apart. In July 2017, we sampled in each station 15 soil cores along an L-shaped transect with 10 m distances between neighbouring cores, corresponding to the biological replicates at the plot scale and aiming to account for microhabitat heterogeneity. This sampling design allowed to account for the local heterogeneity at different spatial scales (from meters to kilometres) and it was a good compromise for covering sufficiently local diversity across groups of varying spatial distributions^57^, while already minimizing spatial autocorrelation as it has been shown for earthworms and bacteria (> 5 m between soil samples^58,59^). Soil corers were cleaned and flame sterilized between each sample collection. Extracellular DNA was then extracted from 15 g as described in^60,61^. Botanical surveys were conducted and consisted of annotating the species present in the vicinity (1 m^2^) of each soil core.

### Laboratory analyses

DNA extractions were conducted at the field on a mobile field unit. PCR, sequencing and soil physico-chemical analyses were performed at the Laboratoire d’Écologie Alpine (LECA) in Grenoble, France. Physicochemical soil properties were quantified from soil cores, including soil organic matter content (%), pH, soil moisture and C (%), N (%) and P content.

DNA extraction, PCR and sequencing negative controls were included in the experiment and used to identify potential contaminants and to control for false positives caused by tag‐switching events. In order to set extracellular DNA (eDNA) free from clay and silica particles, each sample was rotatively shaken for 15 min in a 15 ml saturated phosphate buffer solution (Na_2_HPO_4_; 0.12 M; pH ≈ 8). Two ml of sediment/buffer mixture were then sampled and centrifuged for 10 min at 10,000 g. A 400 µl aliquot of supernatant was recovered and used as starting material for eDNA extraction using NucleoSpin® Soil extraction kit (Macherey–Nagel GmbH, Düren, Germany), following manufacturer’s instructions except skipping the lysis cell step^60^. After elution, DNA extracts were diluted 10 times before being used as template for amplification. Eight negative extraction controls were also performed.

### DNA amplification and sequencing

To assign the sequence reads to their relevant samples after high-throughput sequencing, we added unique eight base-long tags (with at least five differences between each other) to the 5’ end of each primer (modified from^62,63^). DNA amplifications were carried out in a final volume of 20 μl containing 2 μl of DNA sample, 10 μl of AmpliTaq Gold 360 Master Mix 2X (Applied Biosystems™, Foster City, CA, USA), 2 μl of primers mix at initial concentration of 5 μM of each primer and 0.16 μl of Bovine Serum Albumin. A total of 10 PCR negative and six positive PCR controls were included. Each sample (including all controls) was amplified in quadruplicate. Eukaryotes, Fungi and Protists were targeted using the respective DNA markers: Euka02 (18S rRNA gene), Fung02 (ITS1) and Bact01 (16S rRNA gene) described in^20^. PCR thermocycling conditions were as follow: after an initial step of 10 min at 95 °C, the mixtures underwent 45 cycles of 30 s at 95 °C, 30 s at 57–55–45 °C (Bact01, Fung02, Euka02, respectively) and 60 s at 72 °C, followed by a final elongation at 72 °C for 7 min. The amplification success was checked using capillary electrophoresis (QIAxcel System; Qiagen). PCR products were mixed in an equi-volume way (15 µl each) and 8 aliquots of 100 µl of the resulting mix were then purified using MinElute Purification kit (Qiagen GmbH, Hilden, Germany). Purified products were then pooled together before sequencing. This later was performed by pair-end sequencing on Illumina HiSeq 2000 platform (2*125 for Euka02, and 2*250 for both Bact01 and Fung02) at Fasteris, Geneva, Switzerland.

### Bioinformatics

Sequences from the three libraries were pre-processed using the OBITools software^64^. Forward and reverse paired-end reads were assembled based on their overlapping 3’-end sequences, demultiplexed and dereplicated. We then removed sequences with low paired‐end alignment scores, singletons, short sequences and sequences containing ambiguous bases, as well as PCR errors using the *obiclean* command. Molecular Operational Taxonomic Units were built by clustering sequences at 97% of similarity using SUMACLUST^65^. Taxonomic annotations were performed with the SILVAngs pipeline (Quast et al. 2013), using the SILVA version 132 for Bact02 and Euka01. For Fung02 and Euka01 (only metazoa), we used the *ecotag* command from the OBITools, and the EMBL database version 136. Taxonomic annotations with > 75% identities were retained. Cross-sample contaminations and reagent contaminants were removed on the basis of negative and empty controls, and dysfunctional PCRs were detected and removed following the procedures described in^66^ with the metabaR R package^67,68^. For each marker, non-targeted taxa were eliminated. For Euka01 marker, we also excluded MOTUs identified as fungi, plants, and non-soil animals. After curation, PCR replicates were pooled together into samples. Only remaining common samples between the three MOTU tables were retained (n = 86). Number of reads, MOTUs, PCR replicates and samples before and after the curation process are available in Supplementary Table 2.

### Soil food webs

Using current knowledge on soil organisms, we classified the MOTUs, based on their taxonomic annotations, into 9 broad trophic classes, using a classic soil food web backbone (e.g.^22,27^). These trophic classes included primary consumers, decomposers, phytophagous or plant parasites, mycorrhizal fungi, bacterivores, fungivores, omnivores, predators and animal parasites (Fig. 2a). Next, we defined 37 finer trophic groups by separating phylogenetic distant groups that could have a different set of prey/predators (e.g., bacterivore mites and bacterivore nematodes) or groups differing in their resources acquisition strategy (e.g. different types of mycorrhiza and saprotrophs). The definition on the trophic groups was made in accordance with the information available and the taxonomic resolution of the marker (Fig. 2b, Supplementary Fig. 1, Supplementary Table 1). For example, we kept collembola as a unique trophic group because the marker Euka02 was not resolutive enough to assign the MOTUs of this group to the family level, which was needed to a finer trophic classification. We kept both levels of resolution for the analyses, i.e., trophic class and trophic group. The databases used for the taxonomic assignment were FUNGuild^69^ for fungi, FAPROTAX^70^ for bacteria, NEMAguild^69^ and Nemaplex (http://nemaplex.ucdavis.edu/) for nematodes, and the main references used included^71^ for protists (and^72^ for cercozoa), and^73^ for heterotrophic bacteria (i.e. copiotrophic and oligotrophic classification). The main taxonomic clades composing the trophic classes and groups are in Supplementary Table 3. Specific criteria used to define the trophic classes and groups for each kingdom are in Supplementary Table 4. A table for each kingdom including the list of taxa, the trophic groups assignment, the taxonomic level of assignment and the references or databases used is available on Supplementary files.

The MOTU diversity of each trophic group was estimated per sample using the Shannon diversity (i.e. the exponential of the Shannon entropy) since this is a relevant measure for eDNA data^74^. In eDNA metabarcoding studies, changes in the abundance/biomass of an individual taxon may be inferred, in some extents, from changes in their relative abundances across samples, although this correspondence can be noised by different biological or technical factors (reviewed in^20^). However, some taxon can exhibit higher gene copies than others, making these changes in relative abundance more difficult to compare across groups contrary to other abundance standardized measures such as biomass. Relative abundances were thus estimated using a double-transformation. First, the total read counts of each trophic group were converted to proportions within a sample, and second, the resulting proportions were standardized by the largest observed proportion across all samples for each trophic group. Relative abundance of each group varied from 0 (absent) to 1 (largest observed diversity/proportion), allowing to obtain comparable measures across groups. Relative abundances of trophic classes were calculated by summing the relative abundances of the trophic group included in the trophic class^31^.

The *metaweb*, which contains the potential trophic interactions of the soil food webs of the system under study, was built for trophic classes and trophic groups^45^. Trophic links between trophic groups and trophic classes were added based on the main feeding preferences. Some constraints were added when assigning the trophic links between trophic groups based on (1) the organisms size, i.e. predators fed only on smaller preys, with some exceptions like animal parasites and omnivore nematodes that can eat larger preys, and macroorganisms did not interact with microorganisms, (2) habitat differentiation, i.e. strict plant endoparasites (i.e. protists) were not considered as prey of other free living predators, and (3) feeding preferences, e.g. fungivores fed only on saprotrophic fungi and Ectomycorrhizal, which are preferred to arbuscular mycorrhizal fungi^75^. The complete *metaweb* of trophic groups can be found in Supplementary Fig. 1 and the adjacency matrix is available in Supplementary files. Resource nodes were added to the food web representations with a structural purpose and corresponded to the main resources of the soil food web, i.e., sunlight, organic matter and plants, but were excluded from the diversity analyses, because the aim was to quantify the diversity of organisms within the soil food web. Differences in resources and plant composition between undamaged and defoliated forests were evaluated aside with multivariate analyses (see below). The *metaweb* was then used to characterize the composition and structure of the local soil food webs based on the trophic classes or groups detected locally in each soil sample (n = 86), assuming that classes or groups present locally interact as in the *metaweb*. For the local soil food webs at the trophic group resolution, nodes corresponded to the local relative abundance of the groups and links were binary (i.e., present or absent) assuming an interaction when the two groups concerned were present. For the trophic class resolution, nodes corresponded to the sum of the relative abundances of the trophic groups inside the trophic class and the links were weighted by the probability of interaction between two classes given the links between their respective trophic groups and the relative abundances of these groups as a proxy for the probability of an encounter^31^.

### Statistical analyses

Differences in resources and plant composition between undamaged and defoliated forests were evaluated with multivariate analyses. A correspondence analysis was run to evaluate the differences in plant community composition. Plant communities from undamaged forests were mostly associated with ericaceous dwarf shrubs such as *Empetrum nigrum* and *Vaccinium* spp., but also of other shrubs and herbs in Kirkenes, e.g., *Salix* sp., *Betula nana*, *Equisetum* sp. (Supplementary Fig. 2). In defoliated forests plant composition was more variable among samples, including several species of grass and herbs, such as the dominant *Avenella flexuosa*. For the soil physico-chemical characteristics that we measured, the first two axes of a Principal Component Analysis explained 74.7% of the variance. The first axis was related to soil organic matter (SOM) and the second axis was related to the litter quality (measured with the C/N ratio) and inversely to soil acidity (i.e. pH) (Supplementary Fig. 3). Samples from defoliated forests were related to higher values of SOM, C, N, P and pH and lower C/N values.

To assess the effect of moth defoliation on MOTU diversity and the relative abundance of the trophic groups, a multilevel linear model was applied separately to each trophic group using the function ‘stan_lmer’ from the R package Rstanarm^76^ with the default priors. In each model, a fixed effect for defoliation was included as a dummy variable (0 corresponding to the undamaged forest and 1 to the defoliated forests). To account for the structure of the sampling design, i.e. soil cores clustered within stations and stations clustered within areas, we added a nested random term for stations within area to the intercept, where station was a factor with 8 levels and area a factor with 2 levels. Note that random factors allowed for borrowing information from each station and area, and that using a Bayesian approach led to non-zero estimates of area and station random effects, contrary to approaches using REML. Even if we suspected that the effect of defoliation could vary between the areas due to the contrasting habitat characteristics of Kirkenes and Tana, preliminary analyses showed that the effect was similar for both areas (i.e. the coefficient of the interaction between area and defoliation was small and 95% CI widely overlapped with 0 for most groups). MOTU diversity was standardized by the largest diversity observed across samples for each trophic group, to obtain comparable effect sizes across groups. A Yeo-Johnson transformation was applied to the relative abundances to improve the distribution of the residuals. Each model was run with 4 parallel MCMC chains with 15,000 iterations each. Model convergence was assessed visually and by checking Rhat < 1.10 for all the parameters. The normality of residuals was evaluated visually by using quantile–quantile (Q–Q) plots, and residuals were plotted against fitted values to assess outliers or influential values.

To study changes in the structure of local food webs, we estimated network diversity indices using the R package econetwork^31^. It allows computing several diversity indices on groups and link abundances using a viewpoint parameter that control the importance given to low *vs.* high relative abundances. We used a measure of dissimilarity of node and link compositions at different resolutions (trophic group and trophic class) to analyse whether there was a change in the structure of local soil food webs due to defoliation. A mixed multivariate distance matrix regression was then run using the dissimilarity matrix as the response, including a dummy variable for defoliation as a predictor and accounting for the nested sampling design as a random effect using the R package MDMR^77^. Local diversity (α-diversity) was estimated as the generalised mean of local diversity within each category of defoliation (i.e. defoliated forest and undamaged forest) within each area (Tana and Kirkenes). Both network local diversity and dissimilarity were computed using 1 as viewpoint parameter (eta in the package). Using this value of parameter, local diversity is the exponential of Shannon entropy. Figures 2, 3, 4, 5 were made using the R software (R 3.6.3)^68^.

## Supplementary Information

Supplementary Information 1.

Supplementary Information 2.
