# Supplementary material for: Cascading effects of moth outbreaks on subarctic soil food webs
Source: Sci Rep. 2021 Jul 23;11:15054. doi: 10.1038/s41598-021-94227-z (PMC8302651; doi:10.1038/s41598-021-94227-z)
Supplement: Supplementary file 1 — Supplementary Information 1. [file 41598_2021_94227_MOESM1_ESM.docx]

**Supplementary Figures and Tables**

**
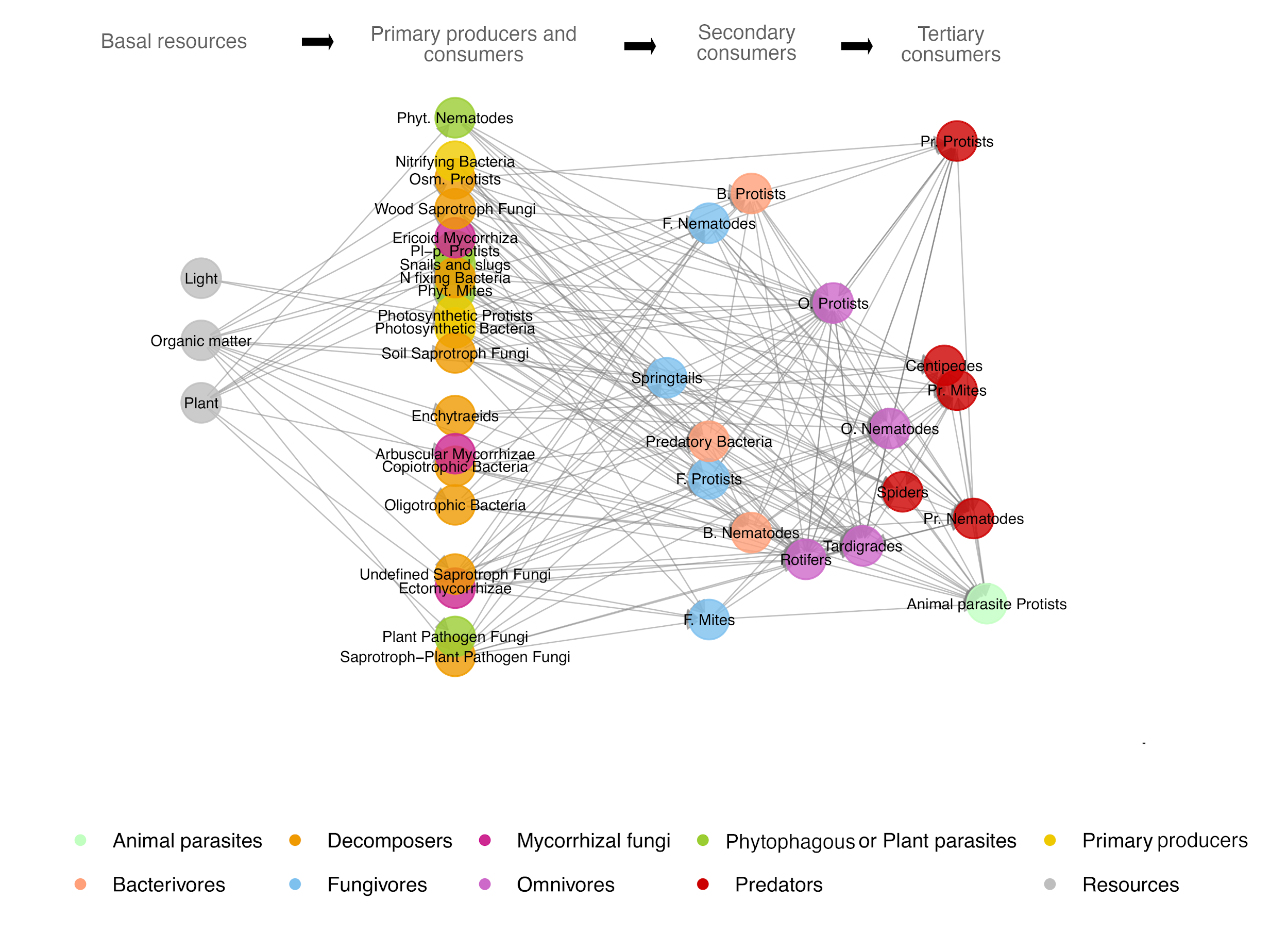
**

**Supplementary Figure 1. *Metaweb* of the trophic groups included in the study.** It contains the potential trophic interactions for the studied system with all the trophic groups (nodes) present at their maximum relative abundance (max.weigth=1). Trophic interactions (links) are binary (i.e. present or absent). Colours correspond to the trophic classes. The nodes are distributed horizontally based on their trophic level from the left (basal levels) to the right (higher levels).


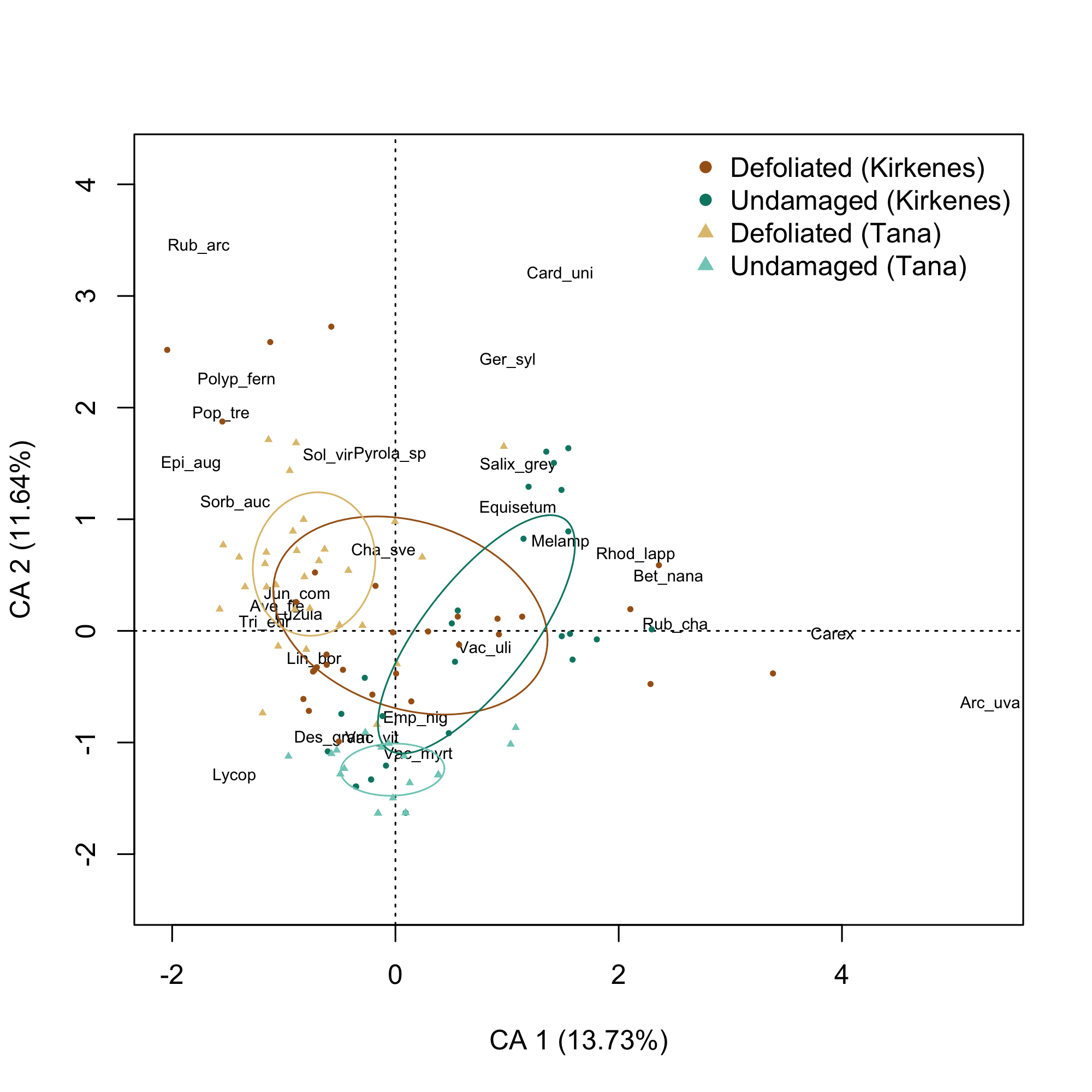


**Supplementary Figure 2. Correspondence analysis of the plant community composition in defoliated and undamaged forests.** The analysis was done on presence/absence data of plant species sampled in a botanical survey in the vicinity (1 m^2^) of each soil core. The first two axes of the correspondence analysis are shown. Ellipses were drawn for samples from defoliated and undamaged forests of both areasTana and Kirkenes. Plant species or group of species code: Arc_uva = *Arctostaphylos uva-ursi*, Ave_fle = *Avenella flexuosa*, Bet_nana= *Betula nana*, Card_uni = *Carduus* spp., Carex= *Carex* sp., Cha_sve = *Chamaepericlymenum suecicum*, Des_gram = Graminae, Emp_nig = *Empetrum nigrum*, Epi_aug = *Epilobium angustifolium*, Equisetum = *Equisetum* spp., Ger_syl = *Geranium sylvaticum*, Jun_com = *Juniperus communis*, Lin_bor = *Linnaea borealis*, Luzula = *Luzula* spp., Lycop = *Lycopodium* spp., Melamp = *Melampyrum pratense*, Polyp_fern = Ferns, Pop_tre = *Populus tremula*, Pyrola_sp = *Pyrola* spp., Rhod_lapp = *Rhododendron lapponicum*, Rub_arc = *Rubus arcticus*, Rub_cha = *Rubus chamaemorus*, Salix_grey = *Salix* spp., Sol_vir = *Solidago virgaurea*, Sorb_auc = *Sorbus aucuparia*, Tri_eur = *Trientalis europaea*, Vac_myrt = *Vaccinium myrtillus,* Vac_uli = *Vaccinium uliginosum*, Vac_vit = *Vaccinium vitis-idaea*.


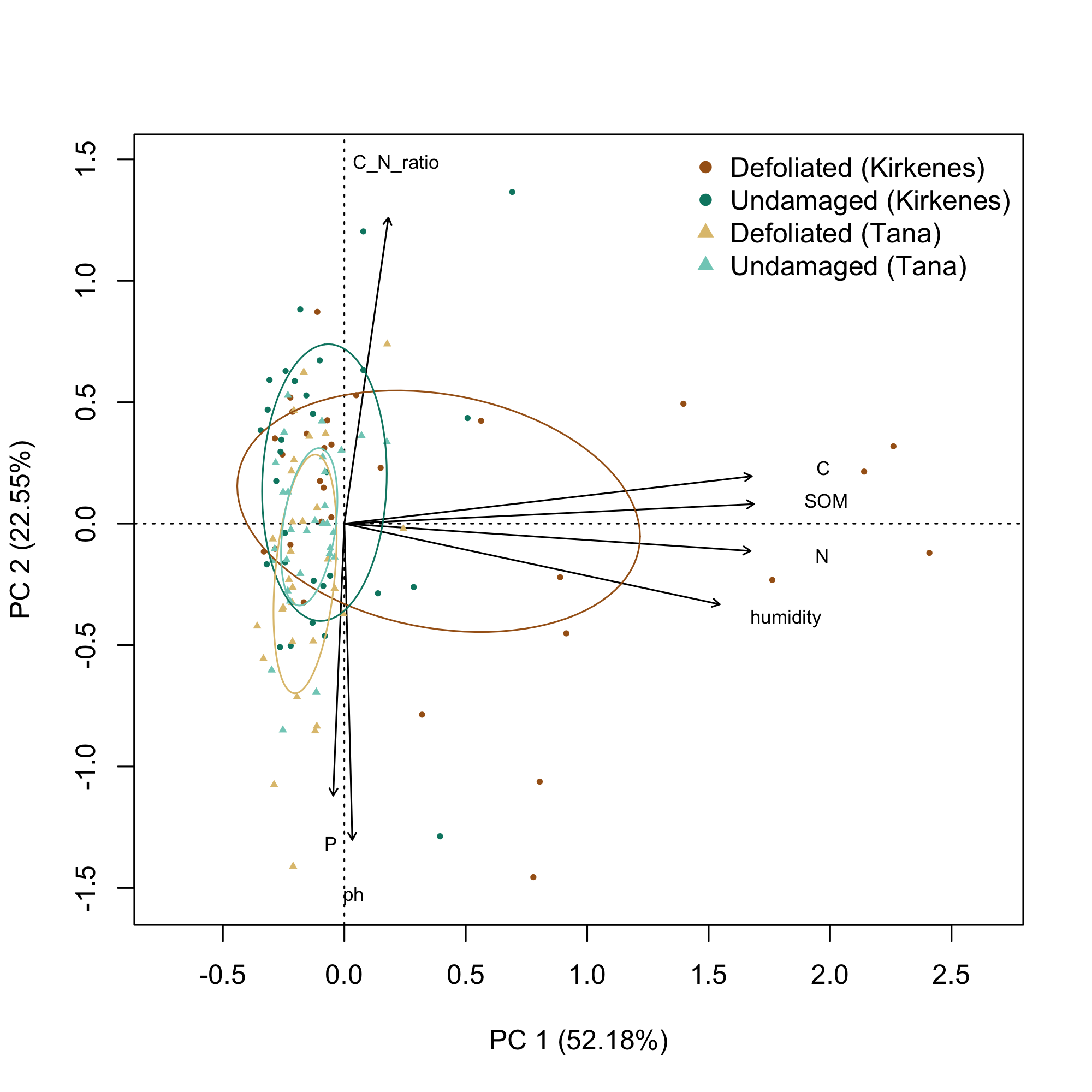


**Supplementary Figure 3. Principal component analysis of the soil physicochemical properties measured in the study site.** The first two axes of the principal component analyses are shown. Ellipses were drawn for samples from defoliated and undamaged forests of both areas Tana and Kirkenes. Soil properties code: SOM= soil organic matter (%), N= total nitrogen (%), C= total carbon (%), P= Phosphate concentration (mg P/kg), humidity = humidity (%), ph = pH (KCl).


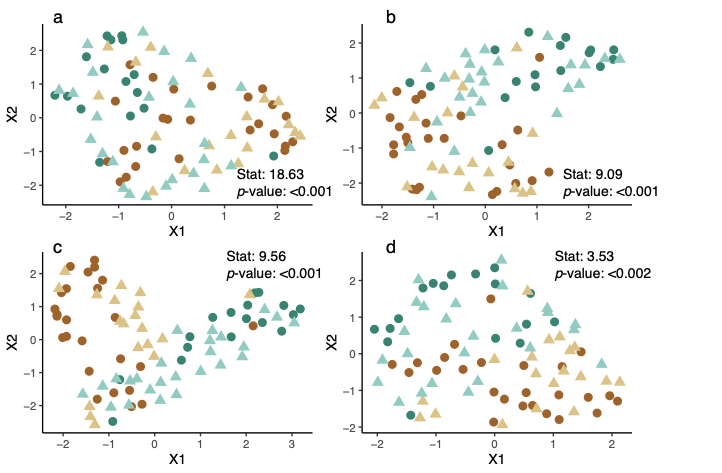


**Supplementary Figure 4. Uniform Manifold Approximation and Projection (UMAP) of the dissimilarities between local food webs.** Upper panels and lower panels correspond to the projection of the local food webs dissimilarities at the trophic class level (a,b) and the trophic group level (c,d), respectively. Colors correspond to undamaged (blue) and defoliated (brown) forest, and shapes to the areas Tana (triangle) and Kirkiness (circle). The statistic and the *p*-value are the result of the mixed multivariate distance matrix regression analysis (MDMR) using defoliation as the explanatory variable and a random term to account for the spatial structure of the sampling design.

**
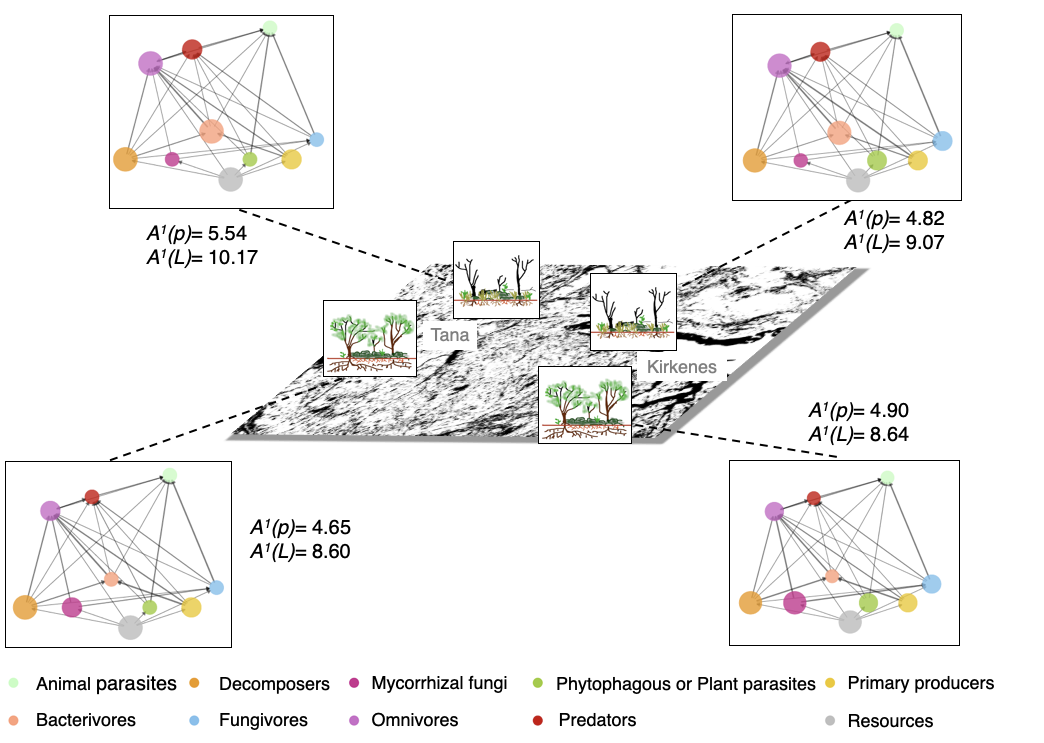
**

**Supplementary Figure 5. Topology and α-diversity of local food webs in undamaged *vs.* defoliated forests.** The values represent the α-diversity of the soil food webs for each area and category of defoliation at the trophic group resolution: A^1^(p) is the diversity in trophic class abundances (nodes) and A^1^(L) the diversity in trophic links abundances (edges) using Shannon diversity. The nodes are weighted by the average local relative abundance of the trophic classes and are distributed vertically based on their trophic level from the bottom (basal levels) to the top (higher levels).

**Supplementary Table 1. Characteristics of trophic classes and trophic groups.** Information of the kingdom, DNA marker, organism size category, total number of reads and total number of MOTUs for each trophic group.

| **Trophic class** | **Trophic group** | **Kingdom** | **Marker** | **size** | **Total reads** | **Total MOTUs** |
| --- | --- | --- | --- | --- | --- | --- |
| Primary producers | Photosynthetic Protists | Protists | Euka01 | Micro | 1054 | 29 |
|  | Photosynthetic Bacteria | Bacteria | Bact02 | Micro | 32395 | 1144 |
|  | Nitrifying Bacteria | Bacteria | Bact02 | Micro | 2696 | 216 |
| Decomposers | Enchytraeids | Metazoa | Euka01 | Meso | 1009858 | 27 |
|  | Osm. Protists | Protists | Euka01 | Micro | 217 | 13 |
|  | Copiotrophic Bacteria | Bacteria | Bact02 | Micro | 1278911 | 16093 |
|  | N fixing Bacteria | Bacteria | Bact02 | Micro | 354 | 42 |
|  | Oligotrophic Bacteria | Bacteria | Bact02 | Micro | 1813303 | 19789 |
|  | Saprotroph-Plant Pathogen Fungi | Fungi | Fung02 | Micro | 34551 | 26 |
|  | Soil Saprotroph Fungi | Fungi | Fung02 | Micro | 1595382 | 247 |
|  | Undefined Saprotroph Fungi | Fungi | Fung02 | Micro | 203282 | 324 |
|  | Wood Saprotroph Fungi | Fungi | Fung02 | Micro | 31961 | 48 |
| Mycorrhizal fungi | Arbuscular Mycorrhizae | Fungi | Fung02 | Micro | 134389 | 63 |
|  | Ectomycorrhizae | Fungi | Fung02 | Micro | 660718 | 232 |
|  | Ericoid Mycorrhiza | Fungi | Fung02 | Micro | 16705 | 16 |
| Phytophagous or Plant parasites | Snails and slugs | Metazoa | Euka01 | Macro | 11203 | 14 |
|  | Phyt. Mites | Metazoa | Euka01 | Meso | 11710 | 2 |
|  | Phyt. Nematodes | Metazoa | Euka01 | Micro | 2775 | 26 |
|  | Pl-p. Protists | Protists | Euka01 | Micro | 2815 | 12 |
|  | Plant Pathogen Fungi | Fungi | Fung02 | Micro | 8516 | 67 |
| Bacterivores | Predatory Bacteria | Bacteria | Bact02 | Micro | 38923 | 1042 |
|  | B. Nematodes | Metazoa | Euka01 | Micro | 51035 | 101 |
|  | B. Protists | Protists | Euka01 | Micro | 78539 | 669 |
| Fungivores | Springtails | Metazoa | Euka01 | Meso | 51084 | 93 |
|  | F. Mites | Metazoa | Euka01 | Meso | 65582 | 62 |
|  | F. Nematodes | Metazoa | Euka01 | Micro | 788 | 16 |
|  | F. Protists | Protists | Euka01 | Micro | 683 | 14 |
| Omnivores | Rotifers | Metazoa | Euka01 | Micro | 5250 | 33 |
|  | O. Protists | Protists | Euka01 | Micro | 231316 | 1231 |
|  | Tardigrades | Metazoa | Euka01 | Micro | 12429 | 30 |
|  | O. Nematodes | Metazoa | Euka01 | Micro | 4977 | 5 |
| Predators | Centipedes | Metazoa | Euka01 | Macro | 12649 | 35 |
|  | Spiders | Metazoa | Euka01 | Macro | 5572 | 27 |
|  | Pr. Protists | Protists | Euka01 | Micro | 138783 | 535 |
|  | Pr. Mites | Metazoa | Euka01 | Meso | 351001 | 101 |
|  | Pr. Nematodes | Metazoa | Euka01 | Micro | 1158 | 12 |
| Animal parasites | Animal parasite Protists | Protists | Euka01 | Micro | 9208 | 176 |

**Supplementary Table 2.** Number of reads, unique sequences (or MOTUs), PCR replicates and samples of each DNA marker before and after each cleaning step. Value reported in the OBITools steps correspond to all the sequences and reads present in both samples and controls, and value reported in the R pipeline correspond only to the sequences from the samples. Replicates correspond to the PCR replicates and samples to the biological samples after pooling PCR replicates.

| **Bioinformatic tool** | **Filtering steps** | **Fung02** | | **Euka02** | | **Bact01** | |
| --- | --- | --- | --- | --- | --- | --- | --- |
|  |  | **MOTUs*** | **reads** | **MOTUs*** | **reads** | **MOTUs*** | **reads** |
| **OBITools** | **Raw** | 27422302 | 65544738 | 1379001 | 20283197 | 5616123 | 30271045 |
|  | **Common filter (length, singletons)** | 284817 | 8132983 | 191724 | 12802290 | 598404 | 7049411 |
|  | **obiclean** | 66605 | 5828675 | 43635 | 12802290 | 355721 | 5902013 |
| **metabaR R package** | **Initial number** | 60954 | 5406842 | 39723 | 12076134 | 334404 | 5457704 |
|  | **Cross-sample contaminants removal** | 60895 | 5267227 | 39654 | 11717233 | 334257 | 5325472 |
|  | **MOTU clustering 97%** | 48155 | 5267227 | 15932 | 11717233 | 69346 | 5325472 |
|  | **Removal of contaminants and putative chimeras** | 3063 | 4471331 | 9195 | 11509820 | 67174 | 5287686 |
|  | **Remove dysfunctional (outliers) PCRs** | 2932 | 4190572 | 8655 | 9927100 | 59608 | 4478161 |
|  | **Remove everything that is not from the target taxa** | 2703 | 4155727 | 8655 | 9927100 | 51817 | 4451801 |
|  |  | **Replicates** | **Samples** | **Replicates** | **Samples** | **Replicates** | **Samples** |
|  | **Initial number** | 480 | 120 | 480 | 120 | 480 | 120 |
|  | **Number after curation** | 307 | 107 | 295 | 101 | 326 | 109 |
|  | **Number of lost** | 173 | 13 | 185 | 19 | 154 | 11 |
|  | **Average number of MOTUS after curation (±sd)** | 76.0 (56) | 114 (82) | 338 (193) | 612 (314) | 2137 (1457) | 4186 (2449) |
|  | **Average number of reads after curation (±sd)** | 13650  (13369) | 38839  (34693) | 33651  (30784) | 98288  (77364) | 13737  (13298) | 40842  (34722) |

*before MOTU clustering this corresponds to the number of unique sequences

**Supplementary Table 3.** Clades to which belong soil organisms within trophic groups in the study site (at phyla, class, order, family or genus level). Rounded relative abundance of reads and proportion of MOTUs were calculated within each trophic group. Note that some clades can be repeated across trophic groups, but the statistics correspond to the subset of the clade assigned to the corresponding group. ‘Others’ correspond to a group of taxa with different taxonomic resolutions. The complete list of taxa assigned to trophic groups is available at the dryad repository.

| **Trophic class** | **Trophic group** | **Clade** | **Relative abundance (%)** | **Proportion of MOTUs (%)** |
| --- | --- | --- | --- | --- |
| Primary producers | Photosynthetic Bacteria | Chloroflexi | 98.9 | 93.38 |
|  |  | Cyanobacteria | 0.55 | 4.68 |
|  |  | Alphaproteobacteria | 0.55 | 1.87 |
|  |  | Gammaproteobacteria | <0.01 | 0.06 |
|  | Photosynthetic Protists | Stramenopiles | 86.92 | 70 |
|  |  | Protalveolata | 6.12 | 6.67 |
|  |  | Excavata | 6.05 | 13.33 |
|  |  | Dinoflagellata | 0.91 | 10 |
|  | Nitrifying Bacteria | Nitrospiraceae | 98.64 | 97.05 |
|  |  | Gallionellaceae | 1.01 | 1.48 |
|  |  | Nitrospinaceae | 0.35 | 1.48 |
| Decomposers (Fungi) | Saprotroph-Plant Pathogen Fungi | Agaricomycetes | 100 | 100 |
|  | Soil Saprotroph Fungi | Archaeorhizomycetes | 100 | 99.27 |
|  |  | Leotiomycetes | <0.01 | 0.37 |
|  |  | Microbotryomycetes | <0.01 | 0.37 |
|  | Undefined Saprotroph Fungi | Agaricomycetes | 69.39 | 30.79 |
|  |  | Geoglossomycetes | 11.8 | 3.11 |
|  |  | Leotiomycetes | 7.01 | 20.62 |
|  |  | Sordariomycetes | 4.04 | 13.56 |
|  |  | Others | 2.66 | 7.91 |
|  |  | Eurotiomycetes | 1.7 | 8.76 |
|  |  | Saccharomycetes | 1.53 | 3.95 |
|  |  | Pezizomycetes | 1.27 | 3.39 |
|  |  | Dothideomycetes | 0.54 | 6.5 |
|  |  | Orbiliomycetes | 0.03 | 0.28 |
|  |  | Cystobasidiomycetes | 0.02 | 0.28 |
|  |  | Microbotryomycetes | 0.01 | 0.28 |
|  |  | Tremellomycetes | <0.01 | 0.56 |
|  | Wood Saprotroph Fungi | Agaricomycetes | 98.01 | 70.59 |
|  |  | Orbiliomycetes | 1.71 | 21.57 |
|  |  | Dothideomycetes | 0.28 | 7.84 |
| Decomposers (Bacteria) | Copiotrophic Bacteria | Actinobacteria | 59.03 | 48.37 |
|  |  | Gammaproteobacteria | 28.34 | 29.35 |
|  |  | Bacteroidetes | 7.59 | 13.03 |
|  |  | Gemmatimonadetes | 4.18 | 4.95 |
|  |  | Firmicutes | 0.86 | 4.3 |
|  | N fixing Bacteria | Beijerinckiaceae | 85.43 | 85.71 |
|  |  | Rhodocyclaceae | 11.3 | 7.14 |
|  |  | Heliobacteriaceae | 1.09 | 2.38 |
|  |  | Magnetococcaceae | 1.09 | 2.38 |
|  |  | Nostocaceae | 1.09 | 2.38 |
|  | Oligotrophic Bacteria | Acidobacteria | 55.27 | 49.97 |
|  |  | Alphaproteobacteria | 17.7 | 14.36 |
|  |  | Verrucomicrobia | 11.9 | 8.84 |
|  |  | Deltaproteobacteria | 10.65 | 17.76 |
|  |  | Planctomycetes | 4.43 | 8.82 |
|  |  | Actinobacteria | 0.05 | 0.25 |
| Decomposers  (Metazoa) | Enchytraeids | Enchytraeidae | 100 | 100 |
|  | Osm. Protists | Ciliophora | 100 | 100 |
| Mycorrhizal fungi | Arbuscular Mycorrhizae | Glomeromycetes | 100 | 100 |
|  | Ectomycorrhizae | Agaricomycetes | 99.25 | 96.85 |
|  |  | Pezizomycetes | 0.58 | 2.1 |
|  |  | Sordariomycetes | 0.11 | 0.35 |
|  |  | Dothideomycetes | 0.05 | 0.7 |
|  | Ericoid Mycorrhiza | Leotiomycetes | 100 | 100 |
| Phytophagous or Plant parasites | Phyt. Mites | Penthaleidae | 100 | 100 |
|  | Phyt. Nematodes | Tylenchida | 100 | 100 |
|  | Pl-p. Protists | Stramenopiles | 72.59 | 91.67 |
|  |  | Rhizaria | 27.41 | 8.33 |
|  | Plant Pathogen Fungi | Dothideomycetes | 56.74 | 31.34 |
|  |  | Leotiomycetes | 34.24 | 40.3 |
|  |  | Sordariomycetes | 7.68 | 5.97 |
|  |  | Exobasidiomycetes | 0.9 | 11.94 |
|  |  | Microbotryomycetes | 0.2 | 1.49 |
|  |  | Entorrhizomycetes | 0.16 | 5.97 |
|  |  | Taphrinomycetes | 0.05 | 1.49 |
|  |  | Others | 0.03 | 1.49 |
|  | Snails and slugs | Caenogastropoda | 86.89 | 85.71 |
|  |  | Heterobranchia | 13.11 | 14.29 |
| Bacterivores | B. Nematodes | Triplonchida | 64.23 | 32.04 |
|  |  | Rhabditida | 18.64 | 33.98 |
|  |  | Enoplida | 15.13 | 11.65 |
|  |  | Monhysterida | 1.94 | 19.42 |
|  |  | Desmodorida | 0.05 | 1.94 |
|  |  | Chromadorida | 0.01 | 0.97 |
|  | B. Protists | Rhizaria | 47.91 | 43.77 |
|  |  | Stramenopiles | 24.6 | 21.01 |
|  |  | Amoebozoa | 8.37 | 14.35 |
|  |  | Choanoflagellida | 7.38 | 9.42 |
|  |  | Ciliophora | 7.19 | 6.67 |
|  |  | Filasterea | 3.71 | 2.9 |
|  |  | Cryptophyceae | 0.81 | 1.74 |
|  |  | Excavata | 0.03 | 0.14 |
|  | Predatory Bacteria | Haliangiaceae | 65.73 | 59.71 |
|  |  | Bdellovibrionaceae | 28.5 | 32.99 |
|  |  | Phaselicystidaceae | 5.52 | 6.45 |
|  |  | Burkholderiaceae | 0.17 | 0.51 |
|  |  | Bacteriovoracaceae | 0.09 | 0.34 |
| Fungivores | F. Mites | Oribatida | 99.99 | 97.01 |
|  |  | Astigmata | <0.01 | 1.49 |
|  |  | Others | <0.01 | 1.49 |
|  | F. Nematodes | Tylenchida | 69.43 | 94.12 |
|  |  | Dorylaimida | 30.57 | 5.88 |
|  | F. Protists | Ciliophora | 100 | 100 |
|  | Springtails | Collembola | 100 | 100 |
| Omnivores | O. Nematodes | Dorylaimida | 100 | 100 |
|  | O. Protists | Rhizaria | 57.84 | 58.74 |
|  |  | Stramenopiles | 21.72 | 19.11 |
|  |  | Ciliophora | 15.17 | 13.21 |
|  |  | Incertae Sedis | 4.14 | 3.73 |
|  |  | Choanoflagellida | 0.77 | 3.5 |
|  |  | Amoebozoa | 0.36 | 1.55 |
|  |  | Dinoflagellata | <0.01 | 0.16 |
|  | Rotifers | Monogononta | 85.27 | 85.29 |
|  |  | Bdelloidea | 14.73 | 14.71 |
|  | Tardigrades | Eutardigrada | 100 | 100 |
| Predators | Centipedes | Chilopoda | 100 | 100 |
|  | Pr. Mites | Endeostigmata | 82.23 | 26.36 |
|  |  | Mesostigmata | 12.26 | 35.45 |
|  |  | Trombidiformes | 4.96 | 32.73 |
|  |  | Astigmata | 0.56 | 3.64 |
|  |  | Others | <0.01 | 1.82 |
|  | Pr. Nematodes | Chromadorida | 66.88 | 64.29 |
|  |  | Mononchida | 26.78 | 21.43 |
|  |  | Triplonchida | 6.34 | 14.29 |
|  | Pr. Protists | Ciliophora | 87.23 | 66 |
|  |  | Rhizaria | 6.91 | 21.7 |
|  |  | Nuclearia | 5.37 | 10.31 |
|  |  | Incertae Sedis | 0.24 | 0.18 |
|  |  | Dinoflagellata | 0.24 | 1.27 |
|  |  | Protalveolata | 0.01 | 0.54 |
|  | Spiders | Araneae | 100 | 100 |
| Animal parasites | Animal parasite Protists | Protalveolata | 69.25 | 68.68 |
|  |  | Apicomplexa | 16.17 | 15.93 |
|  |  | Ichthyosporea | 9.1 | 10.44 |
|  |  | Rhizaria | 5.48 | 4.95 |

**Supplementary Table 4.** Databases and criteria used to assign and select the trophic groups are detailed for each kingdom. The percentage of assignment corresponds to the percentage of number of reads and MOTUs that were both assigned and included in the multitrophic network from the total of sequences obtained for each specific kingdom after data curation. The detail on the taxa assigned, the taxonomic level of assignment and specific references are available on the dryad repository.

| **Kingdom** | **Tools** | **Description of the methods and assigning criteria** | **% assigned** |
| --- | --- | --- | --- |
| Fungi | FUNGuild (Nguyen et al. 2016) | Guilds were kept based on the following criteria: (1) reflecting the diversity of broad trophic groups found in fungi (saprotrophs, symbionts and plant pathogens), (2) that could respond differently to disturbances for the fine groups definition, e.g. we distinguish between the different types of resources used by the saprotroph (wood, soil, undefined) and the different types of mycorrhizal fungi because we expected them to respond differently to the resources fluctuation caused by the moth outbreaks, and (3), that together they represented more than 70% of the reads in the dataset. | Reads:  78.3  MOTUs:  42.2 |
| Bacteria | Faprotax (Louca et al. 2016), Literature | Bacteria were divided in Heterotrophic and Primary producers.  - Photosynthetic included all Cyanobacteria, all Chloroflexi and other taxa identified as phototrophs, and not heterotrophs, from Faprotax.  - Bacteria identified in Faprotax as part of the N-cycle (i.e. nitrifying, N-fixing), pathogens (absent in our dataset) or predatory bacteria were considered as different groups. The rest of the taxa was classified as copiotrophs and oligotrophs based on Ho et al. 2017. | Reads:  89.5  MOTUs:  83.2 |
| Protist | Literature | MOTUs of protists were classified in all the 9 broad trophic groups definitions. Protists were considered Eukarvore (i.e. Predator) when feeding mainly on protists but not bacteria, Bacterivores when feeding mainly on bacteria, and Omnivores when feeding on both bacteria and eukaryotes like in Fiore-Dono *et al.* 2019. Only completely phototroph protists were classified as Photosynthetic and mixotrophs were classified based on their heterotrophic diet. We based mainly on Adl. *et al.* 2019 for trophic groups assignments and we complemented with compiled databases ( Fiore-Dono *et al.* 2019 for Cercozoa, and a general database compiled by the authors). For groups presenting very variable feeding modes (e.g. Dinoflagellata, Cilliophora) we avoided doing generalizations at higher taxonomic levels. We remove protists that were exclusively parasites on vertebrates, because vertebrates were not included in our soil food web. | Reads:  81.6  MOTUs:  71.8 |
| Metazoa | NEMAGuild  (Nguyen et al. 2016)  NEMAPLEX  ([http://nemaplex.ucdavis.edu/Uppermnus/topmnu.htm#](http://nemaplex.ucdavis.edu/Uppermnus/topmnu.htm)) Literature | We kept the following phyla including the most representative groups of animals in soil food webs: Annelida (O. Haplotaxida), Arthropoda, Mollusca (C. Gastropoda), Nematoda, Rotifera and Tardigrada. The phylum Arthropoda was divided in the classes: Arachnida (mites and spiders), Collembola, Chilopoda and Insecta. Orders of insects were very poorly represented in the dataset (1 MOTU, few reads) or non resolutive for the marker, thus we excluded them from the analyses. Next, we classified the taxa in the trophic classes. For taxa with conserved trophic behaviour and/or with no enough taxonomic resolution (due to the DNA marker), we did generalizations concerning their trophic group when it was pertinent (e.g. tardigrada, rotifera). Nematodes and mites were classified into the different trophic classes using NEMAGuild, NEMAPLEX, and more literature for specific taxa not represented in the databases. For Nematoda, no generalizations were made at higher taxonomic levels than family because of the variability of feeding habits within an order. For mites, generalizations were made depending on the group. | Reads:  88.7  MOTUs:  59.1 |
